# The Current Status of *Herpesviridae* as Major Human Pathogens: A 10-Year Diagnostic Evaluation in Germany

**DOI:** 10.3390/pathogens15060631

**Published:** 2026-06-13

**Authors:** Lucio Fortelny, Manfred Marschall

**Affiliations:** Harald zur Hausen Institute of Virology, Friedrich-Alexander-Universität Erlangen-Nürnberg (FAU), 91054 Erlangen, Germany; lucio.fortelny@fau.de

**Keywords:** human herpesviruses, medically important viral pathogens, large European tertiary care center, diagnostic evaluation over last 10 years, sample frequencies, positivity rates, specific parameters, assignment of submittals to clinical departments, highlighting the importance of diagnostic tools and prevention

## Abstract

Herpesvirus infections belong to major pathogens in the human population. This study aimed at evaluating diagnostic data for eight human herpesviruses, based on datasets derived from a large European tertiary care center. Specifically, we analyzed 118,692 herpesvirus submittals to the Diagnostic Division of the Virological Institute, University Hospital Erlangen (UKER), Germany, between July 2014 and June 2024. Our points of focus were the following: (i) the frequencies of herpesvirus diagnostic results with positivity rates, (ii) departments representing main sample submitters, (iii) the specific importance of intensive care units (ICUs), (iv) the COVID-19 pandemic period, and (v) distinct properties of sample types. Overall, we are stating the highest frequencies of diagnostic assessment for herpes simplex virus (HSV), human cytomegalovirus (HCMV), and Epstein–Barr virus (EBV) infections, pointing to their dominant relevance for clinical practice. Notably, HCMV submittals (46.6% of total), together with EBV (26.2%) and HSV (15.7), accounted for almost 90% of all herpesviral diagnostic samples during this period. Within these key groups, HCMV, EBV and HSV showed positivity rates of 14.5%, 35.0%, and 18.5%, respectively. Concerning a main input of sample submittals, two departments were predominant in our center, i.e., the Departments of Haematology–Oncology and Anaesthesiology. These included patients under multifold types of treatment associated with an increased risk of herpesvirus reactivation or primary infection. Furthermore, another high portion of submittals was noted for ICUs and external sources. In addition, a numerical, transient increase in herpesvirus diagnostic submittals, from various sources, was shown for the COVID-19 pandemic years (mostly 2021) as compared to other periods. Combined, these data underlined the importance of clinical monitoring of herpesvirus infections, particularly for high-risk patients, and the steady need of improvements in preventive measures, therapeutic options, and safe diagnostic tools.

## 1. Introduction

The importance of current herpesvirus infections is mostly reflected by the continuously recorded data of clinical burden [1]. The number of routine-like diagnostically analyzed patient samples in our Medical Center of UKER are generally focused on the main groups of pathogenic viruses. These include herpesviruses, retroviruses, hepatitis viruses, and respiratory viral infections like influenza, respiratory syncytial, and coronaviruses. Herpesviruses stand out regarding a combination of features, including their wideness of distribution, diversity in clinical outcome, and life-long persistence. The spectrum in pathogenicity of different herpesviruses spans from serious acute forms and highly contagious spread to long-term chronic developments, like tumor association and autoimmune disease, pregnancy-related threats, reactivation in immunocompromised hosts, and apathogenic courses of infection as well [1,2,3,4,5,6]. Thus, it is a matter of fact that, on a global scale, herpesviruses have to be considered as major human pathogens. The situation of long-term infection with herpesviruses carries the risk of becoming pathogenic under specific conditions. Concerning this pronounced variability in pathogenic outcomes, it is a specifically challenging task to differentiate between fresh, latent, and reactivated forms of infections. In particular, Epstein–Barr virus (EBV) and human cytomegalovirus (HCMV) infections help address very important questions in regular diagnostic work [7]. Concerning antiherpesviral therapy, the current repertoire of approved drugs is observed at an advanced and beneficial level; nevertheless, from a clinical point of view, it is still far from satisfactory. Despite the number of clinically approved antiherpesviral treatments, major limitations are observed. These include the frequent appearance of side effects, viral drug resistance formation, a poorly developed basis for combination treatments, and a lack of drug approval for specifically critical situations, like congenital HCMV infection during pregnancy [8]. Thus, an improvement in treatment options, as well as an innovative strategy for novel antiviral targeting and drugs, are of urgent importance. Beyond this aspect, improvements in the strategies of diagnosis and vaccine-based prevention are also a main focus of interest. The current restriction on vaccines, available exclusively against the varicella zoster virus (VZV) in primary or recurrent appearances, awaits additional developments in the field [2,9,10,11]. Concerning diagnostic monitoring, a prevalence of sample submittals and positivity rates for individual herpesviruses was stated by earlier reports [12,13,14,15]. The present study focuses on a 10-year documentation of diagnostic assessments in Bavaria, Germany. The dataset was derived from a large German academic hospital and maximum care center, the University Hospital Erlangen (UKER). As individual patient identifiers were not retained following anonymization, each laboratory submittal constitutes an independent analytical unit. This approach reflects testing-level activity and departmental diagnostic burden, rather than patient-level infection prevalence. We confirmed the major diagnostic key groups, comprising HCMV, EBV and herpes simplex virus (HSV) infections. Data are discussed in the context of the current clinical situation regarding the pathogenic status of herpesvirus infections, as well as options for improved diagnosis, therapy, and prevention.

## 2. Materials and Methods

### 2.1. Study Design and Diagnostic Assays

A retrospective observational study was conducted to characterize the diagnostic epidemiology of herpesvirus infections, including herpes simplex viruses 1 and 2 (HSV-1, HSV-2), varicella zoster virus (VZV), human cytomegalovirus (HCMV), human herpesviruses 6 and 7 (HHV-6, HHV-7), Kaposi sarcoma-associated herpesvirus (KSHV), and Epstein–Barr virus (EBV). All eight herpesviruses were assessed according to in-house developed protocols of diagnostic viral genome-specific polymerase chain reactions (PCRs). All PCRs were quantitative (Real-Time PCRs), except for HHV-7, which had been qualitative during the period 2014–2021, and has also been transitioned to quantitative Real-Time PCR thereafter. Positivity was defined as values exceeding the cut-off of the internal standard of the respective herpesvirus assay system, which also depended on the sample type. The study was based on a laboratory registry of all sequential clinical diagnostic submittals to the University Hospital Erlangen (UKER) covering a 10-year period (from 1 July 2014 to 29 June 2024). Data were derived from the routine clinical submittals to the Diagnostic Division of the Virological Institute Erlangen. Ethical approval was granted retrospectively on 22 April 2026 by the Ethics Committee of the Medical Faculty, Friedrich-Alexander-Universität Erlangen-Nürnberg (FAU) (approval number: [26-125-Br]) in accordance with the Declaration of Helsinki (2013).

### 2.2. Patient Population

All submittals received by the Diagnostic Division of the Virological Institute Erlangen were included, with the exception of those that failed to produce valid results. Invalid samples included all test results derived from procedural irregularity, restricted sample quantities, and other nonsignificant details. The quantity of excluded invalid test results comprised 0.32% of total submittals. The unit of analysis was the individual laboratory submittal. As data were fully anonymized, the linkage of multiple submittals to a single patient was not feasible. Consequently, departments with high-frequency or protocol-driven testing strategies (e.g., transplant units, intensive care settings) may therefore contribute disproportionately to submittal volume, and positivity rates should be interpreted as testing-level metrics, rather than patient-level assessments. This approach was chosen deliberately to capture the full volume and distribution of diagnostic activity over the 10-year study period.

### 2.3. Data Source

The demographic data for the study samples were extracted from the laboratory information system of the Diagnostic Division of the Virological Institute Erlangen. Prior to analysis, we removed all patient-identifying attributes. Age was calculated using the date of birth at the time of sample receipt; the date of birth was then removed. The variables to be analyzed consisted of age, sex, virus target, test result, referring clinical department, and sample type. More detailed information, such as diagnostic indication and the underlying clinical diagnosis, was not available, nor was any clinical history or background information.

### 2.4. Outcomes and Endpoint Definition

The primary objective of the retrospective descriptive analysis was the distribution of herpesviruses (HSV-1, HSV-2, VZV, HCMV, HHV-6, HHV-7, KSHV, EBV). Positivity rates were defined as the ratio of positive to total valid submittals per virus. Secondary objectives include the number of submittals and the positive results per clinical department, well as the distribution of sample types. Positivity rates were also compared between patients treated in the intensive care unit and those treated with regular care. The exploration of this clinical question was not prespecified and emerged first during the course of data analysis; the findings are therefore interpreted as hypothesis-generating. In this context, data from external contributors as well as measurements conducted for research purposes were excluded in order to establish a direct comparison between patients treated in intensive care units and those treated in regular care environments.

### 2.5. Sample Size and Statistical Considerations

HSV-1 and HSV-2 analyses were performed by a multiplex PCR assay with simultaneous detection of both HSV species; these results were consolidated at the submittal level to avoid double-counting, leading to a final dataset comprising a total of 118,692 submittals. Given the retrospective descriptive nature of the study, a formal, preliminary power calculation was not performed. The study’s substantial sample size ensured sufficient statistical power to reliably describe the distribution of herpesviruses, positivity rates, as well as differences between clinical departments, including small effect sizes.

### 2.6. Statistical Analysis

Analysis was primarily conducted using descriptive statistics and included rates for viral distribution, their absolute numbers as well as positivity rates by submitting department, and material types for the individual herpesviruses. Determination of differences in positivity rates among clinical departments was conducted using Fisher’s exact test, which was applied given the presence of low frequency in subgroup comparisons. The significance level was set at *p* < 0.05, and odds ratios with 95% confidence intervals were calculated to quantify effect size. To account for multiple comparisons across the eight herpesviruses, a Benjamini–Hochberg correction was applied to the exploratory multiple tests. Regarding transparency, the unadjusted *p*-values were additionally given. Statistical analysis and figure were performed using R version 4.5.3 (R Foundation for Statistical Computing, Vienna, Austria; https://www.R-project.org/) within the R Studio-integrated development environment (Posit Software, version 2026.05.0, PBC, Boston, MA, USA). Associated Figure representations were performed using GraphPad Prism version 8.4.3 for Windows (GraphPad Software, Boston, MA, USA). Descriptive graphics and tabulations regarding analyses of submittal volumes, rates and frequencies were performed using Microsoft Excel for Microsoft 365 (version 2603, Microsoft Corporation, Redmond, WA, USA).

## 3. Results and Discussion

### 3.1. Overview of Herpesvirus-Specific Submittal of Clinical Samples: 10-Year Dataset Referring to the Diagnostic Division of the Virological Institute Erlangen

A 10-year evaluation of the diagnostic dataset of herpesvirus-specific submittal of clinical samples was performed in order to examine the epidemiology of individual infections. Moreover, we independently assessed the pathogen constellations and sample types with respect to their distribution and clinical relevance. For this purpose, we analyzed a total of 118,692 herpesvirus diagnostic analyses (total volume) to the Diagnostic Division of the Virological Institute, University Hospital Erlangen (UKER; Bavaria, Germany) spanning the period between 2014 and 2024. The most important findings were as follows: (i) HCMV, EBV, and HSV—representing the prototype species of β-, γ-, and α-herpesviruses, respectively—were the most frequently tested; (ii) the highest numbers of analyses were submitted by the Department of Haematology and Oncology, by external submitters, and by the Department of Anaesthesiology; (iii) out of these, the most frequently submitted samples comprised anticoagulant blood (EDTA), bronchoalveolar lavage (BAL), and cerebrospinal fluid (CSF).

**Figure 1:**
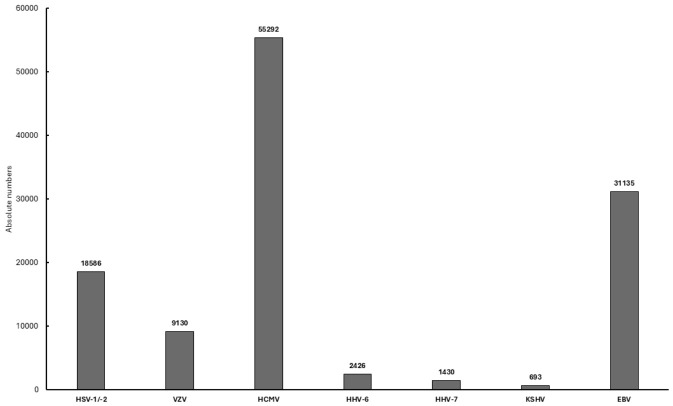
Distribution of total diagnostic assay volume across individual human herpesvirus species. Bar chart showing the absolute number of diagnostic tests performed per virus across all eight herpesviruses (*n* = 118,692 analyses). Herpes simplex virus type 1 (HSV-1); herpes simplex virus type 2 (HSV-2); varicella zoster virus (VZV); human cytomegalovirus (HCMV); human herpesvirus 6 (HHV-6); human herpesvirus 7 (HHV-7); Kaposi sarcoma-associated herpesvirus (KSHV); Epstein–Barr virus (EBV). HSV-1 and HSV-2 were assessed conjointly using type-specific multiplex PCR assay, presented here as a combined single bar.

**Figure 2:**
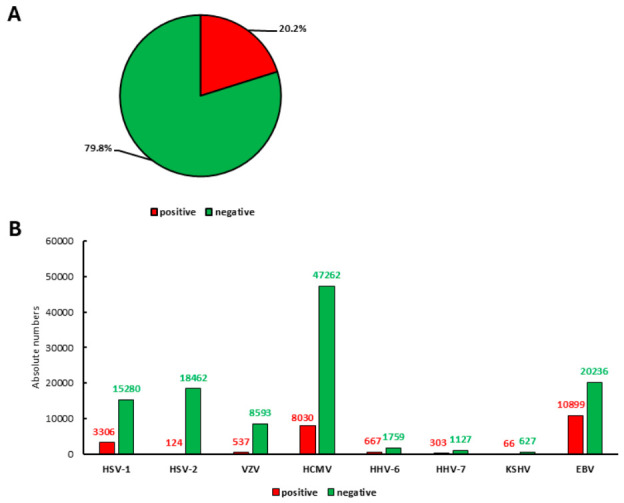
Total positive and negative results in diagnostic sample testing. (**A**) Donut chart showing the distribution of positive (20.2%) and negative (79.8%) results across consolidated submittals (*n* = 118,692); HSV-1 and HSV-2 were determined based on a shared testing protocol, as consolidated at the level of combined submittals. (**B**) Bar chart illustrates the number of positive and negative results from the total number of diagnostic analyses (*n* = 118,692), categorized by herpesvirus species.

**Figure 3:**
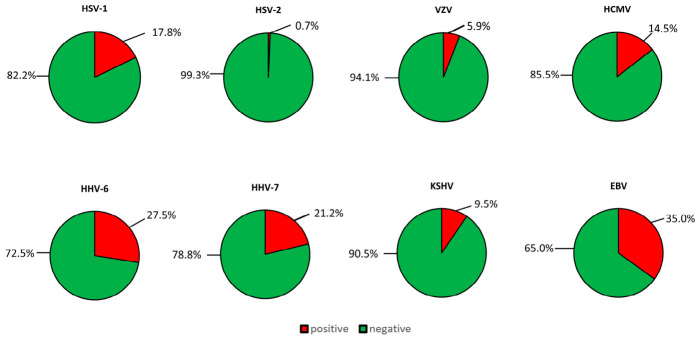
Percentage of positive results per herpesvirus. Donut charts depict the percentages of positive (red) and negative (green) results for each herpesvirus. Positive rates were calculated based on the total assay volume for each virus (see also Figure 1).

A total of 118,692 diagnostic analyses were recorded for eight human herpesviruses, during the 10-year period (total assay volume; HSV-1 and HSV-2 were assessed simultaneously in a combined multiplex PCR assay). Details of the dataset evaluation illustrated that HCMV submittals were dominant (55,292), followed by EBV (31,135) and HSV-1/HSV-2 (18,586). This group accounted for 90% of the total assay volume (Figure 1), completed with smaller sample numbers of VZV (9130), HHV-6 (2426), HHV-7 (1430), and KSHV (693). Overall, 23,932 positive and 113,346 negative results were identified across the total assay volume, corresponding to an overall positivity rate of 20.2% at a consolidated submittal level (Figure 2A). The positivity rates of diagnostic sample testing point to the specific importance of HSV-1, HCMV, and EBV (Figure 2B). Positivity rates were found to be the highest for EBV (35%), followed by HHV-6 (27.5%), HHV-7 (21.2%), and HSV-1 (17.8%). Intermediate percentages were obtained for HCMV (14.5%) and KSHV (9.5%), while VZV (5.9%) and HSV-2 (0.7%) were lower. It has to be emphasized that these positivity rates (Figure 3) were greatly influenced by the selection history of sample submittals; thus, they do not represent general herpesvirus distribution rates in the normal population.

### 3.2. Specification of Viral Samples by Department-Related Submittals and Periods of Time

**Figure 4:**
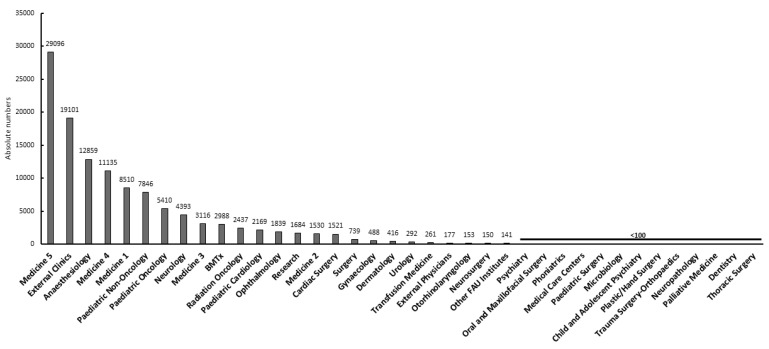
Specification of department-related herpesvirus samples. Bar chart shows the total number of submittals (*n* = 118,692) consecutively received by the Diagnostic Division of the Virological Institute in Erlangen, categorized by submittal origin. Medicine 1, Gastroenterology, Pneumology, and Endocrinology; Medicine 2, Cardiology and Angiology; Medicine 3, Rheumatology and Immunology; Medicine 4, Nephrology and Hypertension; Medicine 5, Haematology and Oncology; BMTx, Center for Stem Cell Transplantation.

**Figure 5:**
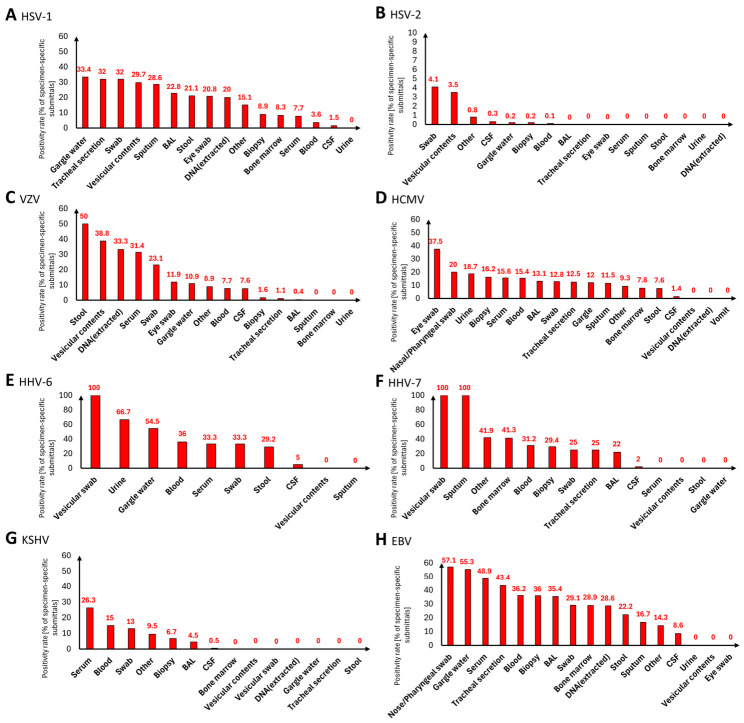
Specification of sample positivity rates referring to submitted materials. Bar charts show the individual number of submitted material types per virus. Abbreviations: Swab, various types of swabs of unspecified origin; BAL, bronchoalveolar lavage; DNA, extracted total DNA of unspecified origin; Biopsy, various types of biopsy material of unspecified origin; Blood, EDTA-treated blood material; CSF, cerebrospinal fluid.

**Figure 6:**
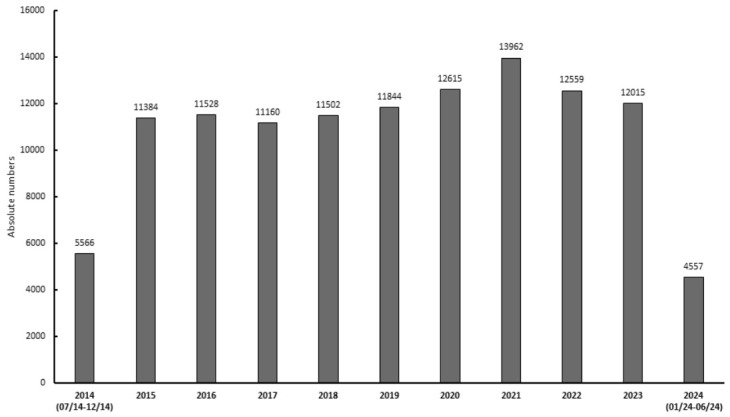
Annual total counts (*n* = 118,692) of herpesvirus sample submittals to the Diagnostic Division of the Virological Institute Erlangen over a 10-year period. Note that the data include partial years (i.e., 1 July to 31 December 2014, and 1 January to 29 June 2024), which therefore account for reduced submittals during this period compared to the complete calendar years (2015–2023).

Concerning the frequency distribution of samples, referring to department-related submittals over time periods, data indicate several specifications (Table 1). Most frequent department-specific submittals, for the three most abundant herpesvirus species, HCMV, EBV, and HSV-1/-2, were recognized for Medicine 5/External Clinics/Medicine 4, or Medicine 5/External Clinics/Paediatric Oncology, or Anaesthesiology/External Clinics/Medicine 4, respectively. A possible limitation of this data evaluation may be seen from the fact that the analysis was conducted at the submittal level, not at the patient level. This may raise the possibility of overcounting bias or distortion of incidence estimates. Nevertheless, the quantitative impact of individual clinical departments is apparent. When considering the entity of all herpesvirus sample submittals, a strong impact was given by the six departments: Medicine 5 (24.5%), External Clinics (16.1%), Anaesthesiology (10.8%), Medicine 4 (9.4%), Medicine 1 (7.2%), and Paediatric Non-Oncology (6.6%) (Figure 4). The distribution of submittals reflects the epidemiological situation of the widespread and disease-specific diversity of herpesviruses [16] in the clinical setting. The majority of submittals came from the field of internal medicine, specifically the Department of Haematology and Oncology (Medicine 5), Department of Nephrology and Hypertension (Medicine 4), and Department of Gastroenterology, Pneumology and Endocrinology (Medicine 3). The reason for this frequency distribution may be attributed to cytotoxic chemotherapy, immunosuppression following kidney transplantation, and inflammatory bowel diseases representing major fields of treatment in these departments. These conditions are likewise well-known risk factors associated with primary infections or reactivation of latent herpesviral diseases [17,18,19]. Notably, the Department of Anaesthesiology has emerged as a leading contributor to herpesvirus submittals. This is consistent with evidence that herpesvirus reactivation frequently occurs in critically ill patients, for example, in the context of septic shock and independently of prior immunosuppression [20]. Such reactivation is followed by significant clinical consequences, as in the case of HCMV being associated with increased mortality in critically ill patients [21]. The detection of HSV in patients with ventilator-associated pneumonia (VAP) is also associated with greater disease severity and a lower prognosis [22]. However, this point should be interpreted carefully, since patients in intensive care units are often subjected to more frequent general monitoring. Moreover, a specification of sample positivity rates, referring to submitted materials, was performed. This illustrated the virus-specific grouping of submitted material types (Figure 5). In regard to the annual annotation of total herpesvirus sample counts, assessed over the period analyzed, we recognized a transient increase in sample submittals by the years 2020, 2021, and 2022 (Figure 6). This period covers the duration of the COVID-19 pandemic. Thus, the reason for this observed increase may be attributed to a substantial rise in diagnostic testing activity. Both the increase in testing activity and in herpesvirus reactivation have been similarly described by others for the pandemic period (reviewed in [23]). Concerning herpesvirus reactivation, at least three factors have been discussed. On the one hand, SARS-CoV-2 gene products may induce reactivation of herpesvirus replication, possibly through the coronavirus interaction with herpesviral or host regulators involved in relevant cellular signaling pathways. On the other hand, SARS-CoV-2 infection causes a cytokine storm leading to severe illness, also indirectly inducing herpesvirus reactivation. In addition, COVID-19-induced immunosuppression, including lymphopenia, hyperinflammation, as well as exhaustion of T cells, may positively modulate herpesvirus infections [23,24].

### 3.3. Quantitative Difference Between Inpatient Intensive Care Units (ICUs) and Regular, Non-ICU Care Units, Concerning Herpesvirus Diagnostic Sample Frequencies and Outcomes

**Figure 7:**
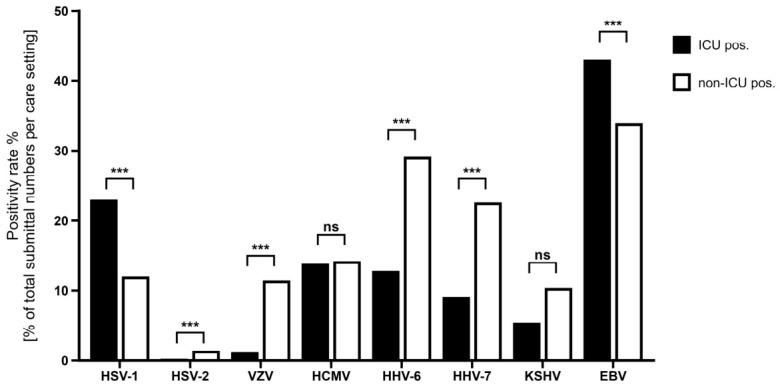
Positivity rate of herpesvirus PCR testing in a comparison between ICU vs. non-ICU inpatient care. The data depict positivity rates (%) of PCR testing for the eight human herpesviruses, broken down by care setting (ICU vs. non-ICU). Each virus was analyzed independently using Fisher’s exact test. Subsequently, Benjamini–Hochberg correction was applied across the eight comparisons to account for false discovery rate at 5%, thus indicating that all statistically significant values remained stable after Benjamini–Hochberg correction, as now indicated in the figure. *** *p* < 0.001; ns, not significant. Absolute case numbers and odds ratios are presented in Table 2.

Analyses were limited exclusively to the inpatient setting; submittals from External Clinics, Medical Care Centers, and External Physicians, as well as research-related submittals, were excluded to ensure clinical homogeneity. Since the virus-specific positivity rate was of particular interest, it was assessed for each virus, based on its respective total number of submittals, in regard of intensive care units (ICUs). It appeared very likely that, especially in ICUs, the medical, economical, and personnel impact may be higher compared to regular care (non-ICUs). In this analytical inpatient cohort, the number of inpatient assays was 112,211 across all eight human herpesviruses, from which 17.2% (19,279) yielded a positive test result. Patients in the ICUs had significantly higher positivity rates for both HSV-1 and EBV infections compared to non-ICUs (Table 2 and Figure 7). Therefore, the most pronounced difference was observed for HSV-1, with ICU 22.8% vs. non-ICU 11.9%; as well as EBV, with ICU 42.8% vs. non-ICU 33.7%. (Table 2.).

The highly significant increase for HSV-1 appeared to be consistent stress-induced virus reactivation in critically ill patients [25]. HSV-1 reactivation is generally associated with a range of local as well systemic stimuli and treatments, such as the administration of corticosteroids, intubation and mechanical ventilation in the intensive care unit while also prolonging mechanical ventilation and extending ICU stay [26,27]. The significantly elevated EBV positivity rates may be similarly explained by viral reactivation upon critical illness. This is consistent with previously reported EBV reactivation rates of up to 54% in critically ill patients in a prospective study, whereby the probability of reactivation increases over the course of the ICU stay in association with a longer duration [28]. Immunological factors associated with increased EBV reactivation in critically ill patients include T-cell exhaustion, which has been linked to higher EBV loads in critically ill patients [29]. This finding particularly indicates that in EBV-specific diagnostic testings, the factor of ICU submittals appears relevant.

Notably, most other herpesviruses showed significantly higher positivity rates in the non-ICU cohort as expected (HSV-2, ICU 0.1% vs. non-ICU 1.3%; VZV, ICU 1.0% vs. non-ICU 11.3%; HHV-6, ICU 12.6% vs. non-ICU 28.9%; and HHV-7, ICU 8.9% vs. non-ICU 22.4%). Interestingly, no significant difference was found for HCMV (ICU 13.7% vs. non-ICU 14.0%) or KSHV (ICU 5.3% vs. non-ICU 10.2%). This lack of significance may be explained, on the one hand, by anti-HCMV prophylaxis of the large portion of transplant patients and, on the other hand, by generally low absolute submittal numbers of KSHV. The lower-ICU-specific positivity rates for VZV, HHV-6, and HHV-7 required specific consideration. Unlike HSV-1 and EBV, for which the elevated ICU rates were suggestively found to be consistent with stress and immunosuppression-driven reactivation, the inverse relationship observed for VZV, HHV-6 and HHV-7 was less readily explained by the illness, whereas two consistent methodological factors could more likely contribute to this finding. Firstly, testing frequencies and clinical indications differed substantially between the care settings. VZV diagnostics in the inpatient non-ICU setting have been predominantly ordered following clinical presentation, such as varicella or dermatomal zoster [30], which may be more frequent in non-ICU compared to ICU. In ICUs, clinical presentation is possibly more often confounded by critical illness because of the severity of other symptoms that may lead to ignore or underrate VZV-specific symptoms. Secondly, differences in patient demographic composition, between non-ICU and ICU settings, may methodologically contribute to the observed lower positivity rates for HHV-6 and HHV-7 in the ICU cohort. Both viruses establish life-long latency following primary infection in early childhood and are subject to clinically relevant reactivation predominantly in immunocompromised patient populations, particularly recipients of hematopoietic stem cell or solid organ transplantation [31] (https://register.awmf.org/de/leitlinien/detail/093-002, accessed on 29 May 2026). Given that post-transplant surveillance is mostly implemented in non-ICU care settings, it is plausible that non-ICU inpatient samples, showing elevated HHV-6 and HHV-7 reactivation, are more disproportionally present in these patient cohorts. Therefore, this demographic overrepresentation might account for the positivity rates observed in the non-ICU setting. These differences in testing frequency and patient demographics, however, could not directly be assessed in a quantitative manner via the present study design and remain hypothetical at this point. Prospective studies with systematic documentation of clinical indication, as well as patient demographics, will be needed to evaluate these aspects.

## 4. Conclusions

The present study, based on the analysis of diagnostic data collected at the University Hospital Erlangen (UKER), provides a clinically relevant overview of herpesvirus infections. The UKER represents a large European university clinical center and maximum care hospital with combined urban–rural target population. Focus was placed on the frequencies of herpesvirus diagnostics with positivity rates, departments representing main sample submitters, the impact of intensive care units, a closer insight into the COVID-19 pandemic years, and additional parameters like sample types. Overall, approximately 90% of all diagnostic submittals in this context were attributed to HCMV, EBV, and HSV, highlighting their dominant clinical burden among human herpesvirus infections. Clinical risk profiles were identified using department-specific submittal patterns; the highest diagnostic volumes were observed in the Departments of Haematology and Oncology, Nephrology and Hypertension, as well as Anaesthesiology. This accumulation of submittals is consistent with immunosuppression, transplantation, and critical illness as the primary driving factors for herpesvirus infection or reactivation. In this context, ICU inpatients showed significantly higher positivity rates for both HSV-1 and EBV compared to non-ICU inpatients, emphasizing the need for targeted diagnostic monitoring in critically ill patients. Based on the identified positivity rates, more intensive monitoring of both HSV-1 and EBV infections in the ICU setting is justifiable, and these identified positivity rates have direct implications for the potential allocation of diagnostic resources. Findings should be interpreted in the context of the analytical framework employed, as patient-level linkage was not feasible due to data anonymization. Thus, positivity rates represent submittal-level data and may therefore be influenced by department-specific testing frequencies. Future prospective surveillance systems should integrate pseudonymized patient identifiers to enable patient-level analysis with even more detailed population-based information. Multicenter analyses at the patient level would solidify potential conclusions and strengthen their epidemiological basis. Nevertheless, the data are strengthening the need of powerful diagnostic monitoring, novel antiviral treatment strategies, as well as new vaccine developments. Due to the clinical burden of human herpesviruses, and according to data of this study, a particular focus should generally be given to HCMV, EBV, and HSV.

## Figures and Tables

**Table 1:** Frequency distribution of herpesviruses across clinical departments *.

Virus	Total Submittals (Absolute Numbers)	Positive (% of Total)	Negative (% of Total)	Most Frequent Department-Specific Submittals	
**HSV-1/-2**	18,586	18.5	81.5	Anaesthesiology External Clinics Medicine 4	4100 3897 2115
**VZV**	9130	5.9	94.1	Anaesthesiology Neurology External Clinics	3771 1612 1032
**HCMV**	55,292	14.5	85.5	Medicine 5 External Clinics Medicine 4	13,075 8591 6674
**HHV-6**	2426	27.5	72.5	Paediatric Non-Oncology Medicine 5 Paediatric Oncology	629 545 431
**HHV-7**	1430	21.2	78.8	Paediatric Non-Oncology Paediatric Oncology Medicine 5	520 297 151
**KSHV**	693	9.5	90.5	Paediatric Non-Oncology Medicine 3 Other FAU institutes	197 95 87
**EBV**	31,135	35.0	65.0	Medicine 5 External Clinics Paediatric Oncology	13,551 5196 2401

* Abbreviations: Department of Medicine 3—Rheumatology and Immunology; Department of Medicine 4—Nephrology and Hypertension; Department of Medicine 5—Haematology and Oncology. Numbers refer to clinical sample submittals to the Diagnostic Division of the Virological Institute Erlangen over the years 2014–2024. HSV-1 and HSV-2 were consolidated at the submittal level (*n* = 18,586); the positivity rate reflects the detection of at least one HSV type per submittal.

**Table 2:** Positivity rates of herpesviruses stratified by care setting (ICU vs. non-ICU inpatient care) *.

Virus	ICU (n)	ICU pos. %	Non-ICU (n)	Non-ICU pos. %	OR (95% CI)	*p*-Value
**HSV-1**	8199	22.8	6458	11.9	2.20 (2.00–2.41)	<0.001
**HSV-2**	8199	0.1	6458	1.3	0.05 (0.02–0.12)	<0.001
**VZV**	4672	1.0	3397	11.3	0.08 (0.06–0.11)	<0.001
**HCMV**	10,338	13.7	34,574	14.0	0.97 (0.91–1.03)	*0.36*
**HHV-6**	269	12.6	1936	28.9	0.36 (0.24–0.52)	<0.001
**HHV-7**	157	8.9	1160	22.4	0.34 (0.18–0.60)	<0.001
**KSHV**	114	5.3	413	10.2	0.49 (0.17–1.20)	*0.14*
**EBV**	2447	42.8	23,420	33.7	1.47 (1.35–1.60)	<0.001

* Fisher’s exact test. A Benjamini–Hochberg correction was applied across the eight comparisons to account for false discovery rate at 5%. All statistically significant values stayed significant after correction; adjusted *p*-values are therefore not displayed separately. ICU, intensive care unit; non-ICU, standard care; pos, positive; OR, odds ratio.

## Data Availability

The original contributions presented in this study are included in the article. Further inquiries can be directed to the corresponding author.
